# Esophageal Ulceration Following the Ingestion of a Single Dose of Doxycycline: A Case Report

**DOI:** 10.7759/cureus.57043

**Published:** 2024-03-27

**Authors:** Mustafa Almayoof, Shahem Abbarh, Areej Mohammed, Bisher Sawaf, Mouhammad J Alawad, Manaf Alobaidy

**Affiliations:** 1 Internal Medicine, Hamad Medical Corporation, Doha, QAT; 2 College of Medicine and Surgery, Almaarefa University, Riyadh, SAU

**Keywords:** case report, esophagitis, esophageal ulcer, drug-induced esophageal injury, doxycycline

## Abstract

Doxycycline is one of the medications that cause drug-induced esophagitis. This condition occurs due to prolonged contact of the medications with the esophageal mucosa, leading to erosion, ulcers, and, in some cases, stricture of the esophagus. Chest pain, dysphagia, and odynophagia are the most common symptoms. Endoscopy is the gold standard for confirming the diagnosis. The treatment consists of stopping the offending medication and starting proton pump inhibitors (PPIs) and sucralfate. Herein, we describe a middle-aged man who presented with severe chest pain, odynophagia, and dysphagia that started two hours after ingesting the first doxycycline pill. An endoscopy showed multiple longitudinal ulcers in the distal esophagus. Symptoms significantly improved after starting a PPI and sucralfate, and feeding was resumed two days later. A follow-up with endoscopy after two months reported completely healed esophageal ulcers. In conclusion, doxycycline-induced esophageal injury is often an underdiagnosed and underreported condition. Physicians and patients should be more aware of doxycycline's detrimental effect on the esophagus, as it can induce esophageal ulceration even after a single dose if not administered properly. Therefore, all patients prescribed oral doxycycline should receive appropriate instructions to minimize this side effect.

## Introduction

Drug-induced esophageal injuries, also known as pill-induced esophagitides, have been reported to be caused by plenty of drugs, with antibiotics contributing to almost half of the cases [1]. The tetracycline class is one of the most commonly implicated antibiotics [1]. Most cases are unreported and/or undetected, and only severe cases seek medical attention, making it difficult to determine the prevalence accurately. Esophageal injuries related to drugs can range from mild erythema and erosions to multiple large deep ulcers. The typical endoscopic appearance of a pill-induced esophageal injury is of discrete ulceration or kissing ulcers with relatively normal surrounding mucosa [2].

The clinical symptoms of doxycycline-induced esophageal ulcerations are similar to other drug-induced esophagitides, including chest pain, dysphagia, and odynophagia [2]. These symptoms commonly start three to 12 days after the initiation of the doxycycline [3]. However, onset has been rarely reported in the literature after a few hours from the first dose [4]. As doxycycline is a commonly prescribed antibiotic, we aim to increase awareness regarding its harmful effect on the esophagus if not administered properly. Herein, we report a middle-aged gentleman who developed multiple esophageal ulcers only two hours after ingesting one doxycycline capsule.

## Case presentation

Our patient is a 48-year-old man with a past medical history of coronary artery disease, status post percutaneous coronary intervention with a stent placement two years before presentation, peptic ulcer disease secondary to Helicobacter pylori (H. pylori) infection twenty years ago, and gastroesophageal reflux disease (GERD) for the last two years. He presented to our hospital with a two-day history of severe substernal/epigastric pain associated with odynophagia and dysphagia for liquids or solids. The patient mentioned that these symptoms started suddenly two hours after taking a doxycycline capsule 100 mg, which his dermatologist prescribed on the same day for acne vulgaris. It is worth mentioning that this was his first dose of doxycycline. He took it two hours after dinner with sips of water and immediately slept in a supine position. Assuming his symptoms were related to doxycycline, he stopped taking it by himself. His other home medicines include clopidogrel, bisoprolol, and as-needed pantoprazole. He denied smoking, alcohol intake, or recent non-steroidal anti-inflammatory drugs (NSAIDs) use.

**Figure 1:**
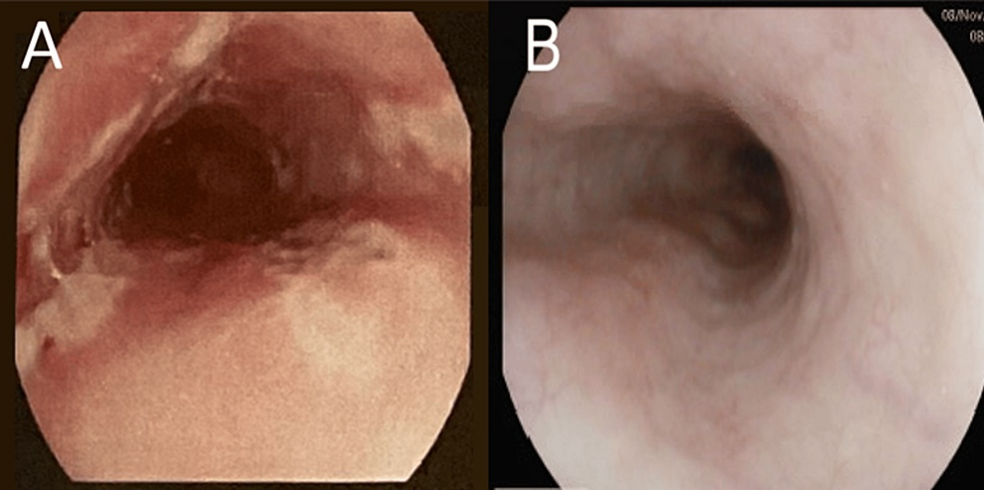
Endoscopic appearance of the esophagus on presentation and during follow-up A: The endoscopic appearance of the esophagus's distal third during admission shows multiple longitudinal ulcers. B: A repeated endoscopy two months after discharge shows normal mucosa with completely healed ulcers.

The patient's vital signs were within normal limits. The physical examination was unremarkable, apart from mild epigastric tenderness. Serial electrocardiogram (ECG) and troponin were negative for myocardial ischemia. Abdominal ultrasound (US) and chest x-ray (CXR) were also unrevealing. An esophagogastroduodenoscopy (EGD) revealed multiple longitudinal ulcers at the distal part of the esophagus and diffuse gastritis (Figure 1A). The biopsy from the esophageal ulcer showed acute esophagitis and was negative for viral inclusions, Barrett's esophagus, fungal elements, or malignancy. H. pylori was negative as well. Notably, a previous EGD, done one year ago for symptomatic GERD, showed only antral gastritis.

The patient was unable to tolerate liquid or oral diets initially. Therefore, he was kept NPO (nothing by mouth) and started on IV pantoprazole and sucralfate. Two days later, he began to improve, and his diet was gradually resumed as tolerated. He was discharged on oral pantoprazole daily, along with as-needed antacids. A follow-up EGD two months later (Figure 1B) was normal, with completely healed esophageal ulcers.

## Discussion

Although pill-induced esophagitis is a common drug-related side effect, physicians may underdiagnose it. The most common drugs contributing to this condition include NSAIDs, bisphosphonate, alendronate, potassium chloride, antihypertensive drugs, and antibiotics (e.g., doxycycline and tetracycline) [2]. Doxycycline is the most well-known antibiotic as the culprit for drug-induced esophagitis [1,2]. Other risk factors for drug-induced esophagitis include being elderly, multiple medications use (e.g., aspirin or alendronate), underlying esophageal motility disease, cardiac enlargement with mid-esophagus compression, and lack of awareness about drug instructions [1]. The pathogenesis is hypothesized to be related to an acidic burn secondary to prolonged direct contact of the medication with the esophageal mucosa [5]. In addition, patient position, the size of the pill, and the amount of water ingested are important determinant risk factors [6]. Doxycycline, in particular, is a relatively large-sized pill with a low pH (pH of <3) [7]. Our patient took a capsule formulation of doxycycline, which has been shown to remain in the esophagus three times longer than its tablet form [3,6].

Based on the previous reports, the onset of the pills-induced esophagitis may develop within a few hours up to 10 days after taking the medication. However, acute presentation, similar to our case, which started after two hours of doxycycline ingestion, has been rarely reported [4]. Patients commonly experience a sudden onset of severe acute retrosternal chest pain or tightness, heartburn, dysphagia, odynophagia, diaphoresis, or vomiting [2]. Hence, this condition may mimic other more serious diseases based on the patient's background, including acute coronary syndrome [8] and esophageal cancer [4]. Complications of delayed diagnosis can present as bleeding, hematoma, ulcer, or even perforation that can eventually lead to death [1,2]. Only 4% of gastrointestinal bleeding in older adults is reportedly due to ulcerative esophagitis, with pill-related bleeding being rarely reported [2,5].

The gold standard for the diagnosis of pill-induced esophagitis is an EGD, which will show esophageal lesions, ranging from erythema to ulcers, in almost all cases [2]. These lesions are frequently located in the middle third of the esophagus [2]. In our patient, the EGD revealed multiple longitudinal ulcers at the distal part of the esophagus and diffuse gastritis.

The treatment consists of stopping the offending agent and starting symptomatic treatment with a proton pump inhibitor (PPI). Analgesics and oral sucralfate may also be added to the treatment plan [3]. In some cases, therapeutic endoscopic intervention was necessary to treat complications of drug-induced esophagitis [1,4]. Our patient and most previously reported cases showed complete healing of the injured mucosa on a follow-up EGD. The prevention of such a complication starts with limiting the unnecessary prescription of the offending drugs. Butt et al. described a case of doxycycline-induced esophagitis, where doxycycline was prescribed for acute bronchitis, a self-limiting condition that does not require antibiotic use [9]. Prevention can also be achieved by adherence to medication instructions on optimal drug intake, including ingestion in an upright position and staying upright for 10-30 minutes after swallowing the medication [8]. In addition, patients should be educated to drink fluids liberally with the doxycycline pill, as advised by the prescribing information of the FDA [10]. 

## Conclusions

Doxycycline-induced esophageal injury is often an underdiagnosed and underreported condition. In this case report, we want to convey two important messages. First, a detailed drug history must be taken from the patients. Second, physicians and patients should be more aware of doxycycline's detrimental effect on the esophagus, as it can induce esophageal ulceration even after a single dose if not administered properly. Therefore, all patients prescribed oral doxycycline should receive appropriate instructions beforehand to minimize this side effect, including ensuring that the pill is taken in an upright position with a large amount of water.

## References

[1] Zografos GN, Georgiadou D, Thomas D, Kaltsas G, Digalakis M (2009). Drug-induced esophagitis. Dis Esophagus.

[2] Kim SH, Jeong JB, Kim JW (2014). Clinical and endoscopic characteristics of drug-induced esophagitis. World J Gastroenterol.

[3] Guo Y, Li HM, Li CX, Zhu WQ, Wang YF, He YH (2019). Oesophageal ulceration in adult patients treated with doxycycline for acne vulgaris. J Int Med Res.

[4] Sasaki Y, Suzuki T, Zai H, Urita Y (2017). Esophageal ulcer associated with inappropriately taken doxycycline: a benign mimicker of esophageal cancer. J Gen Fam Med.

[5] Kikendall JW (1999). Pill esophagitis. J Clin Gastroenterol.

[6] Hey H, Jørgensen F, Sørensen K, Hasselbalch H, Wamberg T (1982). Oesophageal transit of six commonly used tablets and capsules. Br Med J (Clin Res Ed).

[7] Al Rawahi Y, Dutt S (2019). Doxycycline-induced oesophageal ulcer in a teenager: a case report. J Paediatr Child Health.

[8] Roro GM, Folvik G, Louis L, Bane A (2021). Drug-induced esophageal injuries with an atypical presentation mimicking acute coronary syndrome. BMC Gastroenterol.

[9] Butt MA, Peicher M, Nguyen AP, Sheikh AB (2022). Antibiotic stewardship in patients with acute bronchitis: a case report of doxycycline-induced esophagitis. Cureus.

[10] (2022). Food and Drug Administration: Doxycycline Prescribing Information. https://www.accessdata.fda.gov/drugsatfda_docs/label/2022/050641Orig1s032lbl.pdf.

